# Letter to the Editor

**DOI:** 10.3402/jchimp.v3i2.21670

**Published:** 2013-07-05

**Authors:** Humayun J. Chaudhry

**Affiliations:** Federation of State Medical Boards

Dear Editor,

Dr. Paul Kempen's recent guest editorial about MOC (‘Maintenance of Certification – important and to whom?’) includes the inaccurate assertion that the Federation of State Medical Boards (FSMB) ‘envisioned coupling MOC as a requirement for state license renewals as their Maintenance of Licensure (MOL) program’. The FSMB has never advocated that physicians become specialty board certified as a condition of licensure or licensure renewal, and in fact, has very clearly stated publicly the opposite – MOC should *not* be a requirement for MOL. The MOL framework recommended by the FSMB requires neither specialty certification nor Maintenance of Certification (MOC) or Osteopathic Continuous Certification (OCC). MOC or OCC may be an option some physicians wish to use to demonstrate compliance with MOL, but neither program would be required. Stating otherwise does a disservice to *Journal* readers, many of whom are no doubt seeking accurate information about MOL.

The FSMB is working closely with other medical organizations and practicing physicians to ensure that MOL supports a doctor's commitment to lifelong learning, while not being redundant or burdensome. We also recognize that most physicians are already involved in activities that could meet a state's proposed MOL requirements, and we are striving to create a structure for MOL in the United States that takes this into account. We are committed to building a process for demonstrating MOL compliance that is practical, straightforward and efficient – saving time and expense for physicians. We encourage *Journal* readers to visit www.fsmb.org, where they can learn the facts about MOL.

Dr. Kempen also erroneously characterizes my specialty board certification status. I was board certified by the American Board of Internal Medicine in 1996 and by the American Osteopathic Board of Internal Medicine in 2006.

Sincerely,
*Humayun J. Chaudhry, DO, MACP* President and CEO
 Federation of State Medical Boards

